# A Meta-Analysis on Degraded Alpine Grassland Mediated by Climate Factors: Enlightenment for Ecological Restoration

**DOI:** 10.3389/fpls.2021.821954

**Published:** 2022-01-07

**Authors:** Jiale Yu, Lingfan Wan, Guohua Liu, Keming Ma, Hao Cheng, Yu Shen, Yuqing Liu, Xukun Su

**Affiliations:** ^1^State Key Laboratory of Urban and Regional Ecology, Research Center for Eco-environmental Sciences, Chinese Academy of Sciences, Beijing, China; ^2^College of Resources and Environment, University of Chinese Academy of Sciences, Beijing, China

**Keywords:** meta-analysis, climate factors, ecological restoration, Qinghai-Tibet Plateau (QTP), alpine grassland, degradation

## Abstract

Alpine grassland is the main ecosystem on the Qinghai-Tibet Plateau (QTP). Degradation and restoration of alpine grassland are related to ecosystem function and production, livelihood, and wellbeing of local people. Although a large number of studies research degraded alpine grassland, there are debates about degradation patterns of alpine grassland in different areas and widely applicable ecological restoration schemes due to the huge area of the QTP. In this study, we used the meta-analysis method to synthesize 80 individual published studies which were conducted to examine aboveground and underground characteristics in non-degradation (ND), light degradation (LD), moderate degradation (MD), heavy degradation (HD), and extreme degradation (ED) of alpine grassland on the QTP. Results showed that aboveground biomass (AGB), belowground biomass (BGB), Shannon-Wiener index (H′), soil moisture (SM), soil organic carbon (SOC), soil total nitrogen (TN), and available nitrogen (AN) gradually decreased along the degradation gradient, whereas soil bulk density (BD) and soil *pH* gradually increased. In spite of a tendency to soil desertification, losses of other soil nutrients and reduction of enzymes, there was no linear relationship between the variations with degradation gradient. Moreover, the decreasing extent of TN was smaller in areas with higher precipitation and temperature, and the decreasing extent of AGB, SOC, and TN was larger in areas with a higher extent of corresponding variables in the stage of ND during alpine grassland degradation. These findings suggest that in areas with higher precipitation and temperature, reseeding and sward cleavage can be used for restoration on degraded alpine grassland. Fencing and fertilization can be used for alpine grassland restoration in areas with lower precipitation and temperature. Microbial enzymes should not be used to restore degraded alpine grassland on a large scale on the QTP without detailed investigation and analysis. Future studies should pay more attention to the effects of climate factors on degradation processes and specific ecological restoration strategies in different regions of the QTP.

## Introduction

The Qinghai-Tibet Plateau (QTP) is not only the highest plateau in the world (Qiu, 2008) but also an important husbandry production basement (Zhou et al., 2016) and ecological security shelter (Duan et al., 2021) in China and even Asia. Alpine grassland ecosystem covers approximately 60% of the total area of the QTP, which is approximately 2.5 million km^1^ (Dong and Sherman, 2015). It provides not only critical ecosystem services in water regulation, soil conservation, biodiversity maintenance, sand fixation, climate change mitigation, carbon sequestration, and others (Dong and Sherman, 2015; Ding et al., 2016; Ren et al., 2016; Liu et al., 2018; Cao et al., 2020) but also a large number of husbandry products and plant resources required by a human for maintaining social and economic development at local, regional, and global scales (Zhang et al., 2015; Zhuang et al., 2019; Xu et al., 2020). In recent years, due to the impact of global warming and intensified human activities, coupled with unreasonable utilization and management of alpine grassland, nearly half of the alpine grassland is facing degradation (Mao et al., 2008; Dong et al., 2010; Pan et al., 2017b). Alpine grassland degradation will inevitably lead to the further decline of its ecosystem services, which will lead the QTP into a vicious circle of “ecological deterioration and economic poverty,” and ultimately result in great loss of the ecological security shelter and even “ecological disaster” (Zhang, 2006; Liu et al., 2012). The livelihoods of more than 12 million herders living on the QTP will be directly affected by alpine grassland degradation, and the health and well-being of hundreds of millions of people living downstream will also be indirectly affected (Harris, 2010; Dong and Sherman, 2015). Given the seriousness of this situation, our study aims to reveal the processes and driving factors of alpine grassland degradation, providing theoretical support and restoration strategies for preventing alpine grassland degradation and promoting sustainable utilization of alpine grassland ecosystems of QTP.

Due to the aggravation of alpine grassland degradation, not only degradation and restoration of alpine grassland have become a research hotspot but also the demand for ecological restoration engineering with universality, practicability, and operability is becoming more and more urgent (Zhang Q. et al., 2019; Bai et al., 2020; He et al., 2020a,b). In the process of degradation, huge changes have taken place in the vegetation coverage, biomass and biodiversity (Peng et al., 2012; Tang et al., 2015), soil physical and chemical properties (Ma X. et al., 2020; Wen et al., 2020), soil microbial biomass, and enzymes of alpine grassland (Zhang et al., 2017; Zhou et al., 2019). Understanding the processes and stages of alpine grassland degradation and the effects of climate factors and soil properties is the premise of adopting correct restoration strategies (Gao and Li, 2016; Feng et al., 2019; Li et al., 2019). At present, there are numerous studies on alpine grassland degradation, which are mainly divided into two classes, namely, regional research by remote sensing and GIS (Feng et al., 2017; Yu et al., 2017; Chen et al., 2021) and local research by single-site case studies such as field observation and experimental measurement (Li et al., 2014b,^2020^; Jing et al., 2015). Regional research is difficult to describe the mechanisms of degradation due to the lack of field experiments. Local research is difficult to ensure deducing a prevailing reaction type of plant and soil and their interaction due to the huge area of QTP. It is very important to comprehensively analyze the alpine grassland degradation on the QTP by meta-analysis. However, the existing studies using meta-analysis ignore the changes of microbial enzymes in the degradation process and do not consider the effects of climate factors and soil properties (Yan et al., 2020; Zhang W. et al., 2019; Chen et al., 2020; Teng et al., 2020; Liu et al., 2021). Some studies suggest that increasing alpine grassland degradation is significantly affected by climate changes (Huang et al., 2016; Pan et al., 2017b; Ran et al., 2019). It is necessary to conduct a comprehensive analysis to understand the effects of climate factors on degradation processes. There are gaps and doubts on the relationship between soil conditions and alpine grassland degradation on the QTP. The relationships among vegetation, soil, and microbial characteristics with different climate and soil properties in the degradation process of alpine grassland are also not well understood.

Therefore, using the meta-analysis method to study the linkage of vegetation, soil, and microbial characters with different precipitation and temperature in the degradation process of alpine grassland can supplement the shortcomings of the above. We tried to determine the differences of vegetation, soil, and microbial characteristics in the four degradation stages [i.e., light degradation (LD), moderate degradation (MD), heavy degradation (HD), and extreme degradation (ED)] of alpine grassland and the association of climate factors and soil properties of non-degradation (ND) on them. We synthesized to address the following: (1) changes of vegetation, soil, and microbial characteristics in degradation gradients of alpine grassland; (2) effects of climate factors and soil properties on alpine grassland degradation; and (3) implications of ecological restoration strategies to achieve sustainable development of alpine grassland on the QTP.

## Materials and Methods

### Data Collection

Peer-reviewed papers published were searched from the Web of Science and China National Knowledge Infrastructure^2^ using the combined keywords “soil,” “degradation or degeneration or degraded,” “alpine grassland or alpine meadow or alpine steppe,” and “Qinghai-Tibetan Plateau or Tibetan Plateau or Tibet” until April 28, 2021. Only studies meeting the following criteria were included in this meta-analysis: (a) degradation stages were clearly stated and consistent with the classification of alpine grassland degradation on the QTP (Supplementary Table 1; Ma et al., 2002); (b) data from paired ND and degraded alpine grassland were reported, and the initial climate conditions, species compositions, and soil properties were similar in the degraded and ND alpine grassland; (c) the study contained at least one of the soil property and microbial variables (Figure 2); (d) the mean, SD, and sample size (*n*) for each variable could be obtained.

**FIGURE 1:**
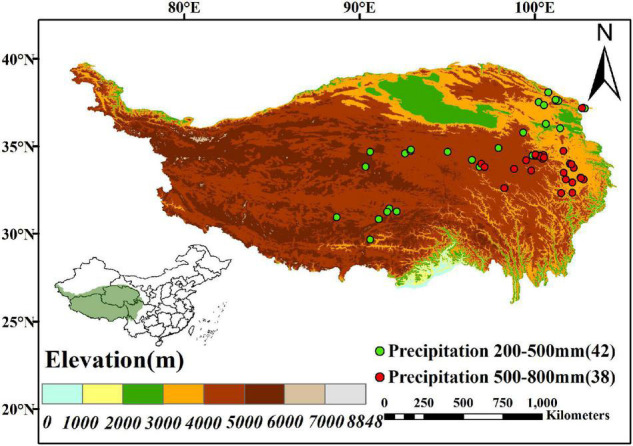
Distribution map of the study site for publications on the Qinghai-Tibet Pl ateau (QTP) of China. Green points represent sites with 200–500 mm mean annual precipitation (MAP) (42 sites), and red points represent sites with 500–800 mm MAP (38 sites).

**FIGURE 2:**
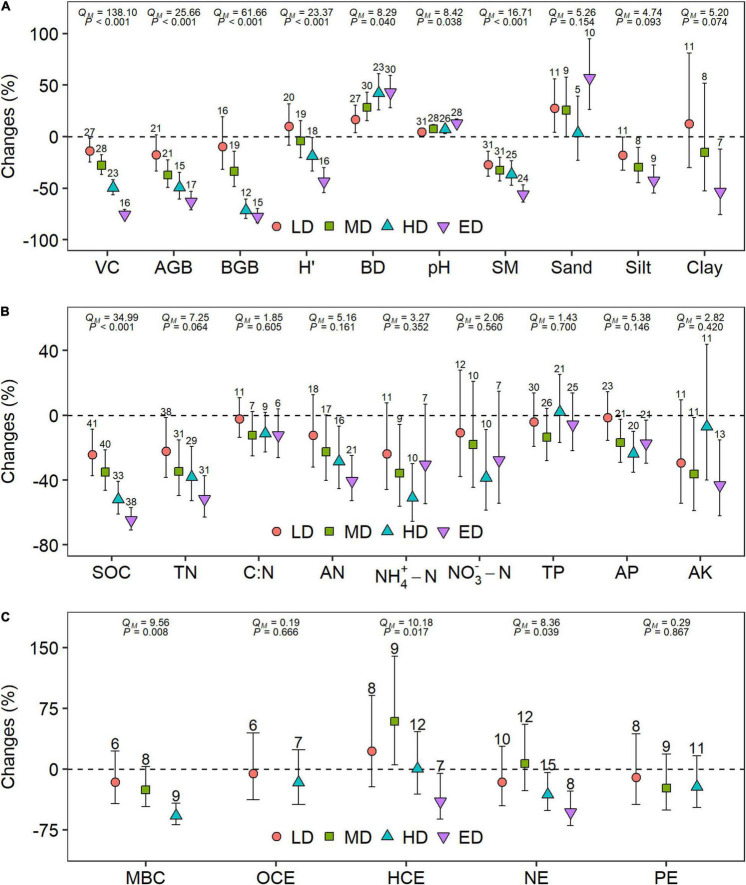
Percent changes of **(A)** plant and six soil property variables, **(B)** soil nutrient variables, and **(C)** microbial variables after grassland degradation across different degradation stages. Weighted means and their 95% CIs of percent changes are given. The numbers at the top of the CIs represent the sample sizes. The significances of degradation stages are tested by the omnibus test (QM). LD, light degradation; MD, moderate degradation; HD, heavy degradation; ED, extreme degradation; VC, vegetation cover; AGB, aboveground biomass; BGB, belowground biomass; H’, Shannon-Wiener index; BD, soil bulk density; SM, soil moisture; SOC, soil organic carbon (C); TN, total nitrogen (N); AN, available N; *NH*_4_-N, ammonium N; $NO_{3}^{-}$-N, nitrate N; TP, total phosphorus (P); AP, available P; AK, available potassium; MBC, microbial biomass C; OCE, oxidative C-cycling enzymes; HCE, hydrolytic C-cycling enzymes; NE, N-cycling enzymes; PE, P-cycling enzymes. Source data are provided as a Source Data file.

A total of 209 paired observations from 80 publications were calculated in our meta-analysis (Figure 1 and Supplementary Appendix). We extracted four plant variables, 15 soil property variables, and 12 microbial variables from the papers. Except for microbial biomass carbon (MBC), we also recorded 11 types of enzymes related to soil C, N, and P cycling. They included oxidative C-cycling enzymes: (1) phenol oxidase and (2) peroxidase; hydrolytic C-cycling enzymes: (3) α-1,4-glucosidase, (4) β-1,4-glucosidase, (5) cellobiohydrolase, (6) β-1,4-xylosidase, and (7) invertase; *N*-cycling enzymes: (8) *N*-acetyl-β-glucosaminidase, (9) L-leucine aminopeptidase, and (10) urease; and P-cycling enzymes: (11) phosphatase. These enzymes are good proxies of soil biogeochemical cycling (Kandeler et al., 1999; German et al., 2011) and are widely used to estimate the ecosystem functionality of microbial communities (Delgado-Baquerizo et al., 2016; Zhou et al., 2020). We used the digitizing software WebPlotDigitizer to extract results presented in figures (Burda et al., 2017). The location, mean annual temperature (MAT), and mean annual precipitation (MAP) were also gathered and recorded. If climate factors were absent from the source papers, we extracted MAT and MAP from the relevant latitude and longitude of the global climate layers of WorldClim (1 km^2^ spatial resolution^2^). The Map of China was provided by the “National Tibetan Plateau Data Center.”^3^ The digital elevation model (DEM) data were downloaded from the United States Geological Survey (USGS) at 30-m spatial resolution.^4^

### Data Analysis

In this meta-analysis, the effects of alpine grassland degradation were calculated using the natural log of the response ratio (RR) (Hedges et al., 1999). The *RR* and its corresponding pooled variance (*v*) were calculated as follows:


(1) $$\text{RR}=\ln\left(\frac{X_{\text{d}}}{X_{\text{nd}}}\right)$$

(2) $$v=\frac{{\text{SD}}_{\text{nd}}^{2}}{n_{\text{nd}}X_{\text{nd}}^{2}}+\frac{{\text{SD}}_{\text{d}}^{2}}{n_{\text{d}}{\text{X}}_{\text{d}}^{2}}$$

where *X*_nd_ and *X*_d_ are the mean values of a specific variable in the ND and degraded alpine grassland, respectively; *n*_nd_ and *n*_d_ are the sample sizes for the ND and degraded groups, respectively; and SD_nd_ and SD_d_ are the SDs for the ND and degraded groups, respectively. In the studies that neither SD nor SE was reported, we assigned the SD as 1/10 of the mean (Zhou et al., 2017). For ease of understanding, we synthesized 11 enzymes into 4 categories (i.e., oxidative C-cycling enzymes, hydrolytic C-cycling enzymes, N-cycling enzymes, and P-cycling enzymes). In addition, the random-effect model was used to calculate RRs of oxidative C-cycling enzymes, hydrolytic C-cycling enzymes, and N-cycling enzymes for each observation (Zhou et al., 2020). The mixed-effect model was used to calculate the weighted *RR* (RR_++_) and corresponding 95% CIs of target variables with a moderator of degradation stages. We performed the omnibus test (*Q*_M_) to test whether the responses differed among different degradation stages. When the *Q*_M_ values were significant (*P* < 0.05), the responses among stages were different. For ease of understanding, the weighted *RR* and its corresponding 95% CI were transformed to the percentage change calculated by the following formula: (*e*^RR^_++_ - 1) × 100%. The groups with the small sample size (<5) were removed in these analyses. Moreover, publication bias within each variable was assessed using a regression test for funnel plot asymmetry (Egger et al., 1997). Our results showed that most variables had no publication bias (Supplementary Table 2). The possible publication bias within several variables would not affect our results because Rosenberg’s fail-safe number indicated that these results were robust (Rosenberg, 2005).

We used single meta-regression models to quantify how effects of degradation varied depending on climate factors (i.e., MAT and MAP) and corresponding variables of non-degradation grassland [i.e., aboveground biomass (AGB), belowground biomass (BGB), soil organic carbon (SOC), soil total nitrogen (TN), and microbial biomass carbon (MBC)]. Finally, we conducted a Pearson correlation analysis to investigate the relationships among RRs of all variables. All statistical analyses were conducted using R software version 4.0.0 (R Development Core Team, 2020).

## Results

### Changes of Vegetation, Soil, and Microbial Variables Along the Degradation Gradient

For four plant variables, the losses escalated with the degradation of alpine grasslands and differed significantly (*P* < 0.001) between the different degradation stages (Figure 2A). However, only vegetation cover significantly decreased at all four degradation stages. The reduction in AGB and BGB was not significant at the stage of LD, and the Shannon-Wiener index only showed a significant reduction at the stages of HD and ED.

Soil bulk density (BD) and soil *pH* significantly increased at all four degradation stages with significantly (*P* < 0.05) higher at the stage of ED than that at the stage of LD (Figure 2B). SM significantly decreased at all four degradation stages with significantly (*P* < 0.001) lower at the stage of ED than that at the stage of LD (Figure 2A). Changes in soil texture showed no significant difference (*P* > 0.05) between the different degradation stages (Figure 2A). Sand content showed a significant increase at the stages of LD and ED. Silt content showed a significant decrease at the stages of HD and ED. In addition, clay content showed a significant decrease at the stage of ED. SOC and TN significantly decreased at all four degradation stages and the reduction both gradually increased along the degradation gradient (Figure 2B). The losses of SOC were significantly higher at the stage of HD and ED compared to those at the stage of LD (*P* < 0.001). The difference in TN losses was not significant (*P* > 0.05) between the different degradation stages. Soil C:N ratio and TP showed no significant changes in all degradation stages (*P* > 0.05). Other nutrients also had a trend to decline, but the variations were rarely consistent at different degradation stages.

For microbial variables, MBC significantly decreased at the stage of HD (Figure 2C). Hydrolytic C-cycling enzymes significantly increased at the stage of MD and significantly decreased at the stage of ED. N-cycling enzymes significantly decreased at the stages of HD and ED. In other degradation stages, the microbial variables did not show significant changes.

### Driving Factors of Alpine Grassland Degradation

**FIGURE 3:**
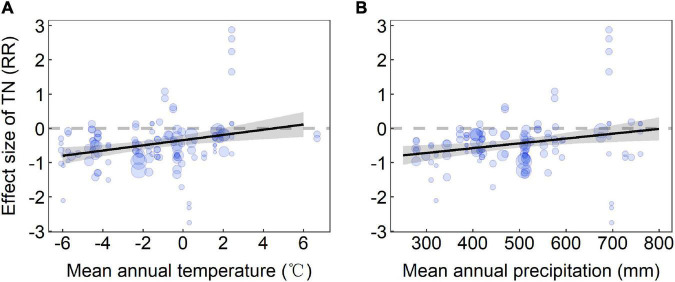
Change in soil total nitrogen with **(A)** mean annual temperature and **(B)** MAP. RR, response ratio. *RR* = 0, dashed gray line; predicted mean effect size (with 95% CI in gray), black lines. The size of data points (in blue) is proportional to the sampling variance. Results were obtained with single meta-regressions.

**FIGURE 4:**
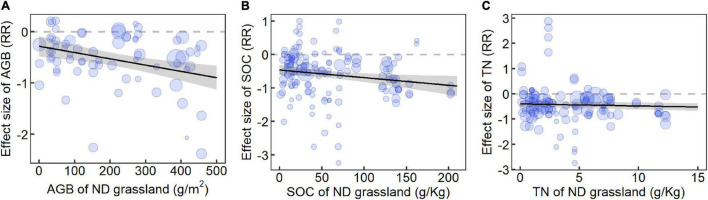
Changes in **(A)** aboveground biomass, **(B)** soil organic carbon, and **(C)** soil total nitrogen with corresponding variables of non-degradation grassland. *RR*, response ratio. *RR* = 0, dashed gray line; predicted mean effect size (with 95% CI in gray), black lines. The size of data points (in blue) is proportional to the sampling variance. Results were obtained with single meta-regressions.

**FIGURE 5:**
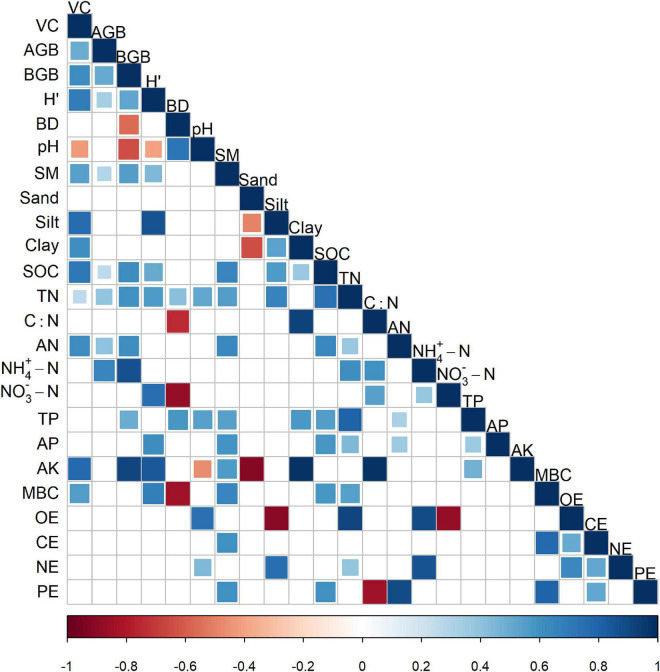
Relationships of the effect sizes of each variable with others. Square size and colors represent Pearson correlations. Only significant results with a sufficient sample size (*n* > 4) were displayed.

The meta-regression results showed that the reduction of TN was smaller in areas with higher MAT and MAP (Figure 3). The increase of soil *pH* was larger in areas with higher MAT and MAP (Supplementary Figures 1a,b). Moreover, the decreasing extent in SM was larger in areas with higher MAP (Supplementary Figure 1c). The losses of AGB, SOC, and TN were larger in areas with a higher extent of corresponding variables in the stage of ND (Figure 4). Correlation analysis showed that most RRs of variables were significantly positively related to each other (Figure 5). Especially, the RRs of vegetation cover, BGB, and Shannon-Wiener index were negatively related to the *RR* of soil *pH*.

## Discussion

### Processes and Mechanisms in Vegetation and Soil Degradation

With more variables included in our study, our results reveal more comprehensive plant, soil, and microbial changes during degradation processes than previous meta-analyses (Zhang W. et al., 2019; Teng et al., 2020; Liu et al., 2021). At the stage of LD, the insignificant changes of AGB and BGB were probably due to the increasing biomass of forbs, which could cancel out the decline in the biomass of graminoids and sedges (Wang et al., 2009; Zhang W. et al., 2019). The Shannon-Wiener index only significantly decreased in the stages of HD and ED, which implied that the light and moderate environmental disturbance might have a neutral effect on vegetation communities (Wang et al., 2009). With the proportion of poisonous plants and the disturbance intensity increasing, plant community structure gradually deteriorates, leading to the reduction of plant diversity (Li et al., 2014a). As shown in correlation analysis, the deterioration of soil properties was closely associated with alpine grassland degradation. Following the loss of vegetation cover, the soil surface losses its protection and undergoes erosion from wind and water, resulting in the increase of sand content and decline in silt and clay content (Xu et al., 2019). Moreover, as topsoil was eroded, subsoil with higher *pH* values would be exposed and form new topsoil (Ma X. et al., 2020), so that the *pH* values also escalated with degradation stages. In fact, accompanied by vegetation degradation, changes in soil properties would further influence other properties. For instance, the shift of plant composition might enlarge the plant transpiration as the big leaves of forbs might exacerbate water loss (Peng et al., 2018). The increase in sand content and decline in SOC also have negative effects on soil water retention, resulting in the reduction of SM with degradation (Pan et al., 2017a; Dai et al., 2021). In addition, the increase of soil BD is also the combined effect of the compaction from livestock trampling, and the loss of SOC as SOM is loose and porous (Ruehlmann and Koerschens, 2009).

Previous studies have proposed some mechanisms to explain the SOC loss with alpine grassland degradation, which mainly focuses on two aspects: (1) the biological and ecological processes and (2) the physical processes. First, the significant decrease of AGB and BGB will directly reduce the input of C to the soil. The previous study has proved that forbs can lead to higher fine root decomposition and enhance the SOC mineralization compared with grass (Fornara et al., 2009). Therefore, changes in the plant community during degradation may lead to a higher rate of SOC mineralization. Our results also showed that the C-cycling enzyme activities would not decrease except the stage of ED, indicating that microbial metabolism related to SOC mineralization would not be hampered when the degradation intensity was not severe. Second, wind and water erosion directly remove SOM. In addition, the physical protection of SOM can be broken by external disturbances such as livestock trampling, which stimulates decomposition (Dong et al., 2020b). Our results showed that the soil C:N ratio did not have significant changes in all degeneration stages, indicating that the N (nitrogen) mostly came from organic sources and was closely related to SOC. Therefore, mechanisms for the loss of SOC could be considered to interpret the loss of soil TN with degradation. Nevertheless, available nitrogen (AN) did not significantly decrease in the stage of LD and MD, possibly resulting from the decline of graminoids which are N-extravagant plants (Turner et al., 2007). Other nutrients mostly had no significant changes at different degradation stages, implying that the influence of degradation on them might be slight and negligible. For microbial variables, our results showed that enzyme activities only significantly decreased in the stage of HD or ED, indicating that HD or ED might be the turning point for enzymes during alpine grassland degradation as the biological or abiotic factors (such as microbial biomass and *pH*) change (Ma W. et al., 2020; Wu et al., 2020).

### The Effect of Climate Factors and Soil Properties on Degradation Processes

Due to the importance of climate factors and soil properties on degradation, we analyzed the relationship between climate factors and soil properties of ND and degraded alpine grassland to improve our understanding of alpine grassland degradation and restoration on the QTP. We found that climatic factors played an important role in alpine grassland degradation, especially for TN, which was consistent with previous studies (Pan et al., 2017b). We also found that AGB, SOC, and TN of ND had significant effects on AGB, SOC, and TN at different degradation stages (Huang et al., 2021). Through these analyses, we can understand the effects of environmental factors during degradation and provide a scientific theory for grassland restoration and sustainable management.

Our results showed that in areas with higher precipitation and temperature, the loss of TN decreased in the alpine grassland degradation process. If that will be the case, there is a “hydrothermal-hole effect” (Shang et al., 2006) in the process of alpine grassland degradation on the QTP. Theoretically, the alpine grassland degradation of QTP is often accompanied by vegetation reduction and soil property changes, and alpine grassland is bare if degradation continues. A large amount of water and heat is lost through the bare land, which makes the “vegetation-soil-microorganism” system of alpine grassland unstable. It accelerates the area of grassland degradation, the invasion of poisonous weeds, and the loss of soil nutrients, thus aggravating the degree of degradation (Dong et al., 2020a). These effects are caused by water and heat entering the atmosphere due to bare soil, which in turn lead to increased degradation, air exchanges of water, and heat decrease with precipitation and temperature increasing. With precipitation and temperature increasing, the effects of the “hydrothermal-hole effect” on degraded alpine grassland can be slowed down, and the loss of TN is also reduced.

The more AGB, SOC, and TN are lost in areas with the more AGB, SOC, and TN of ND alpine grassland during alpine grassland degradation. Studies have shown that the alpine steppe and alpine meadow ecosystems on the QTP have the strongest resistance stability, the weakest resilience stability, and the longest recovery time (Huang et al., 2021). The species-poor systems are more resistant to perturbation than the species-rich systems and show a larger initial resilience following a perturbation (Pfisterer and Schmid, 2002). Our results showed that it was a positive correlation between effect sizes of species richness and SOC, TN, AP, NO_3_^–^-N, and AK. Previous research results showed that there was a positive correlation between most soil properties and species richness in high-altitude natural grassland (Wang et al., 2014). Therefore, the alpine grassland with better soil conditions degenerates more seriously in degradation processes, which is more vulnerable to grassland degradation and needs more recovery time. For example, most of the extreme degradation of “black soil land” occurs in the Sanjiangyuan National Park with better soil properties of ND, but it rarely appears in the alpine grassland in the Northern Tibetan region of China with poor soil properties of ND. We speculate that grassland degradation may be more serious where the resistance stability is better. In areas with better soil properties, the lower the resilience stability of the grassland ecosystem leads to AGB, SOC, and TN loss increasing during alpine grassland degradation.

### Restoration Strategies of Degraded Alpine Grassland

The degradation process is different at various degraded alpine grassland ecosystems, and climate and soil properties play a significant role in the degradation process. According to the comprehensive analysis, our research points out the main ecological restoration directions in different areas on the QTP. In view of the severe degradation of the alpine grassland ecosystem caused by environmental changes and human activities, restoration efforts have been paid more and more attention. In terms of degradation gradient, different restoration measures should be adopted for different degradation areas. “Black soil land” (ED alpine grassland) is difficult to recover without human intervention because it has lost its ability to self-recover (Shang et al., 2006). Artificial planting should be used for restoration in the ED alpine grasslands. In the LD alpine grassland, the soil nutrients and excellent pasture species have not been lost a lot so fencing enclosure for a short time is adopted for restoration. Some scholars believe that 3–4 years is a suitable time for alpine grassland fencing on the QTP (Zhu et al., 2016). In terms of climate factors, fencing and fertilization can be used for alpine grassland restoration in lower precipitation and temperature areas (Dong et al., 2010). Reseeding and sward cleavage can be used for restoration on degraded alpine grassland in higher precipitation and temperature areas (He et al., 2020b). In terms of soil conditions, not only the fragile alpine grassland needs to be protected but also the alpine grassland with the strongest resistance and stability needs more protection due to the longest recovery time (Andrade et al., 2015). If alpine grassland degradation occurs, the alpine meadow is more difficult to recover than the alpine steppe. Microbial enzymes should not be used to restore degraded alpine grassland in large areas on the QTP due to the huge area and diversity of the QTP. If ecological restoration is carried out from the perspective of microbial enzymes, detailed investigation and research must be carried out (Zhang et al., 2021). Grazing management is the first priority for the restoration of degraded alpine grassland (Jiang et al., 2020). According to the degradation degree of alpine grassland, fencing with different lengths of time should be used. In artificial cultivation, species based on different functional traits and different functional groups are selected to make better use of resources (Weisser et al., 2017).

## Conclusion

Our meta-analysis quantitatively assessed the effects of alpine grassland degradation on vegetation, soil, and microbial variables on the QTP. We presented more plentiful data for soil properties and much more information on microbial variables, providing a crucial reference for future studies on alpine grassland degradation. Results showed that the loss of TN tended to alleviate in areas with higher precipitation and temperature during alpine grassland degradation, but the losses of AGB, SOC, and TN tended to aggravate in areas with more contents of ND alpine grassland. These findings improve our understanding of how climate variables and AGB, SOC, and TN of ND alpine grassland regulate degradation stages at a large scale. Ecological restoration of degraded alpine grassland can be carried out by means of fence and fertilization in regions with lower precipitation and temperature. Severe degraded alpine grassland can be restored by reseeding and mowing. Grazing management and artificial cultivation are the most widely used and most effective methods. Implementing different restoration methods in alpine grassland with different degradation gradients and different environmental conditions is necessary to achieve targets of ecological restoration on the QTP.

## Data Availability Statement

The datasets presented in this study can be found in online repositories. The names of the repository/repositories and accession number(s) can be found in the article/Supplementary Material.

## Author Contributions

JY and LW conceived the idea, assembled the data, and wrote the first draft of the manuscript. JY did the statistical analyses. GL, KM, and XS advised in the process of manuscript writing. All authors contributed substantially to the interpretation of the results and revision of the manuscript.

## Conflict of Interest

The authors declare that the research was conducted in the absence of any commercial or financial relationships that could be construed as a potential conflict of interest.

## Publisher’s Note

All claims expressed in this article are solely those of the authors and do not necessarily represent those of their affiliated organizations, or those of the publisher, the editors and the reviewers. Any product that may be evaluated in this article, or claim that may be made by its manufacturer, is not guaranteed or endorsed by the publisher.

## Footnotes

1
http://www.worldclim.org/

2
http://www.cnki.net

3
https://data.tpdc.ac.cn/zh-hans/

4
http://earthexplorer.usgs.gov/

## References

[B1] Andrade B. O. Koch C. Boldrini I. I. Velez-Martin E. Hasenack H. Hermann J. M. (2015). Grassland degradation and restoration: a conceptual framework of stages and thresholds illustrated by southern Brazilian grasslands. *Nat. Conserv.* 13 95–104. 10.1016/j.ncon.2015.08.002

[B2] Bai Y. F. Ma L. N. Degen A. A. Rafiq M. K. Kuzyakov Y. Zhao J. X. (2020). Long-term active restoration of extremely degraded alpine grassland accelerated turnover and increased stability of soil carbon. *Glob. Change Biol.* 26 7217–7228. 10.1111/gcb.15361 32974963

[B3] Burda B. U. O’Connor E. A. Webber E. M. Redmond N. Perdue L. A. (2017). Estimating data from figures with a web-based program: considerations for a systematic review. *Res. Synth. Methods* 8 258–262. 10.1002/jrsm.1232 28268241

[B4] Cao W. Wu D. Huang L. Liu L. L. (2020). Spatial and temporal variations and significance identification of ecosystem services in the Sanjiangyuan National Park, China. *Sci. Rep.* 10:13. 10.1038/s41598-020-63137-x 32273561 PMC7145809

[B5] Chen L. T. Jiang L. Jing X. Wang J. L. Shi Y. Chu H. Y. (2021). Above- and belowground biodiversity jointly drive ecosystem stability in natural alpine grasslands on the Tibetan Plateau. *Glob. Ecol. Biogeogr.* 30 1418–1429. 10.1111/geb.13307

[B6] Chen Y. Feng J. G. Yuan X. Zhu B. (2020). Effects of warming on carbon and nitrogen cycling in alpine grassland ecosystems on the Tibetan Plateau: a meta-analysis. *Geoderma* 370:10. 10.1016/j.geoderma.2020.114363

[B7] Dai L. Guo X. Ke X. Du Y. Mang F. Cao G. (2021). The variation in soil water retention of alpine shrub meadow under different degrees of degradation on northeastern Qinghai-Tibetan plateau. *Plant Soil* 458 231–244. 10.1007/s11104-020-04522-3

[B8] Delgado-Baquerizo M. Maestre F. T. Reich P. B. Jeffries T. C. Gaitan J. J. Encinar D. (2016). Microbial diversity drives multifunctionality in terrestrial ecosystems. *Nat. Commun.* 7:10541. 10.1038/ncomms10541 26817514 PMC4738359

[B9] Ding J. Z. Li F. Yang G. B. Chen L. Y. Zhang B. B. Liu L. (2016). The permafrost carbon inventory on the Tibetan Plateau: a new evaluation using deep sediment cores. *Glob. Change Biol.* 22 2688–2701. 10.1111/gcb.13257 26913840

[B10] Dong S. K. Sherman R. (2015). Enhancing the resilience of coupled human and natural systems of alpine rangelands on the Qinghai-Tibetan Plateau. *Rangel. J.* 37 1–3. 10.1071/rj14117

[B11] Dong S. K. Wen L. Zhu L. Li X. Y. (2010). Implication of coupled natural and human systems in sustainable rangeland ecosystem management in HKH region. *Front. Earth Sci.* 4:42–50. 10.1007/s11707-010-0010-z

[B12] Dong S. Shang Z. Gao J. Boone R. B. (2020a). Enhancing sustainability of grassland ecosystems through ecological restoration and grazing management in an era of climate change on Qinghai-Tibetan Plateau. *Agric. Ecosyst. Environ.* 287:106684. 10.1016/j.agee.2019.106684

[B13] Dong S. Zhang J. Li Y. Liu S. Dong Q. Zhou H. (2020b). Effect of grassland degradation on aggregate-associated soil organic carbon of alpine grassland ecosystems in the Qinghai-Tibetan Plateau. *Eur. J. Soil Sci.* 71 69–79. 10.1111/ejss.12835

[B14] Duan H. C. Xue X. Wang T. Kang W. P. Liao J. Liu S. L. (2021). Spatial and temporal differences in alpine meadow, alpine steppe and all vegetation of the Qinghai-Tibetan Plateau and their responses to climate change. *Remote Sens.* 13:669. 10.3390/rs13040669

[B15] Egger M. Smith G. D. Schneider M. Minder C. (1997). Bias in meta-analysis detected by a simple, graphical test. *Br. Med. J.* 315 629–634. 10.1136/bmj.315.7109.629 9310563 PMC2127453

[B16] Feng X. Qu J. J. Fan Q. B. Tan L. H. An Z. S. (2019). Characteristics of desertification and short-term effectiveness of differing treatments on shifting sand dune stabilization in an Alpine Rangeland. *Int. J. Environ. Res. Public Health* 16:4968. 10.3390/ijerph16244968 31817807 PMC6949889

[B17] Feng Y. F. Wu J. S. Zhang J. Zhang X. Z. Song C. Q. (2017). Identifying the relative contributions of climate and grazing to both direction and magnitude of alpine grassland productivity dynamics from 1993 to 2011 on the Northern Tibetan Plateau. *Remote Sens.* 9:136. 10.3390/rs9020136

[B18] Fornara D. A. Tilman D. Hobbie S. E. (2009). Linkages between plant functional composition, fine root processes and potential soil N mineralization rates. *J. Ecol.* 97 48–56. 10.1111/j.1365-2745.2008.01453.x

[B19] Gao J. Li X. L. (2016). Degradation of frigid swampy meadows on the Qinghai-Tibet Plateau: current status and future directions of research. *Progr. Phys. Geogr. Earth Environ.* 40 794–810. 10.1177/0309133316659283

[B20] German D. P. Weintraub M. N. Grandy A. S. Lauber C. L. Rinkes Z. L. Allison S. D. (2011). Optimization of hydrolytic and oxidative enzyme methods for ecosystem studies. *Soil Biol. Biochem.* 43 1387–1397. 10.1016/j.soilbio.2011.03.017

[B21] Harris R. B. (2010). Rangeland degradation on the Qinghai-Tibetan plateau: a review of the evidence of its magnitude and causes. *J. Arid Environ.* 74 1–12. 10.1016/j.jaridenv.2009.06.014

[B22] He J. Bu H. Hu X. Feng Y. Li S. Zhu J. (2020a). Close-to-nature restoration of degraded alpine grasslands: theoretical basis and technical approach. *Chin. Sci. Bull.* 65 3898–3908.

[B23] He J. Liu Z. Yao T. Sun S. Lu Z. Hu X. (2020b). Analysis of the main constraints and restoration techniques of degraded grassland on the Tibetan Plateau. *Sci. Technol. Rev.* 38 66–80.

[B24] Hedges L. V. Gurevitch J. Curtis P. S. (1999). The meta-analysis of response ratios in experimental ecology. *Ecology* 80 1150–1156.

[B25] Huang K. Zhang Y. J. Zhu J. T. Liu Y. J. Zu J. X. Zhang J. (2016). The influences of climate change and human activities on vegetation dynamics in the Qinghai-Tibet Plateau. *Remote Sens.* 8:876. 10.3390/rs8100876

[B26] Huang W. J. Wang W. Cao M. Fu G. Xia J. Y. Wang Z. X. (2021). Local climate and biodiversity affect the stability of China’s grasslands response to drought. *Sci. Total Environ.* 768:145482. 10.1016/j.scitotenv.2021.145482 33736341

[B27] Jiang S. Feng T. Liu G. He J. (2020). A bibliometric analysis of the application of grassland ecological restoration technology. *Pratacultural Sci.* 37 685–702.

[B28] Jing X. Sanders N. J. Shi Y. Chu H. Y. Classen A. T. Zhao K. (2015). The links between ecosystem multifunctionality and above- and belowground biodiversity are mediated by climate. *Nat. Commun.* 6:8. 10.1038/ncomms9159 26328906 PMC4569729

[B29] Kandeler E. Luxhoi J. Tscherko D. Magid J. (1999). Xylanase, invertase and protease at the soil-litter interface of a loamy sand. *Soil Biol. Biochem.* 31 1171–1179. 10.1016/s0038-0717(99)00035-8

[B30] Li J.-H. Yang G.-J. Wang S.-P. (2020). Vegetation and soil characteristics of degraded alpine meadows on the Qinghai-Tibet Plateau, China: a review. *Yingyong Shengtai Xuebao* 31 2109–2118. 10.13287/j.1001-9332.202006.002 34494765

[B31] Li X. L. Gao J. Zhang J. Wang R. Jin L. Q. Zhou H. K. (2019). Adaptive strategies to overcome challenges in vegetation restoration to coalmine wasteland in a frigid alpine setting. *Catena* 182:104142. 10.1016/j.catena.2019.104142

[B32] Li Y.-Y. Dong S.-K. Liu S. Wang X. Wen L. Wu Y. (2014a). The interaction between poisonous plants and soil quality in response to grassland degradation in the alpine region of the Qinghai-Tibetan Plateau. *Plant Ecol.* 215 809–819. 10.1007/s11258-014-0333-z

[B33] Li Y. Y. Dong S. K. Wen L. Wang X. X. Wu Y. (2014b). Soil carbon and nitrogen pools and their relationship to plant and soil dynamics of degraded and artificially restored grasslands of the Qinghai-Tibetan Plateau. *Geoderma* 213 178–184. 10.1016/j.geoderma.2013.08.022

[B34] Liu J. Milne R. I. Cadotte M. W. Wu Z. Y. Provan J. Zhu G. F. (2018). Protect third pole’s fragile ecosystem. *Science* 362 1368–1368. 10.1126/science.aaw0443 30573620

[B35] Liu X. Long R. Shang Z. (2012). Interactive mechanism of service function of alpine rangeland ecosystems in Qinghai-Tibetan Plateau. *Acta Ecol. Sin.* 32 7688–7697.

[B36] Liu X. Wang Z. Zheng K. Han C. Li L. Sheng H. (2021). Changes in soil carbon and nitrogen stocks following degradation of alpine grasslands on the Qinghai-Tibetan Plateau: a meta-analysis. *Land Degrad. Dev.* 32 1262–1273. 10.1002/ldr.3796

[B37] Ma W. Li G. Wu J. Xu G. Wu J. (2020). Response of soil labile organic carbon fractions and carbon-cycle enzyme activities to vegetation degradation in a wet meadow on the Qinghai-Tibet Plateau. *Geoderma* 377:114565. 10.1016/j.geoderma.2020.114565

[B38] Ma X. Asano M. Tamura K. Zhao R. Nakatsuka H. Wuyunna (2020). Physicochemical properties and micromorphology of degraded alpine meadow soils in the Eastern Qinghai-Tibet Plateau. *Catena* 194:104649. 10.1016/j.catena.2020.104649

[B39] Ma Y. Lang B. Li Q. Shi J. Dong Q. (2002). Study on rehabilitating and rebuilding technologies for degenerated alpine meadow in the Changjiang and Yellow river source region. *Pratacultural Sci.* 19 1–5.

[B40] Mao F. Zhang Y.-H. Hou Y.-Y. Tang S.-H. Lu Z.-G. Zhang J.-H. (2008). Dynamic assessment of grassland degradation in Naqu of northern Tibet. *J. Appl. Ecol.* 19 278–284. 18464632

[B41] Pan T. Hou S. Wu S. Liu Y. Liu Y. Zou X. (2017a). Variation of soil hydraulic properties with alpine grassland degradation in the eastern Tibetan Plateau. *Hydrol. Earth Syst. Sci.* 21 2249–2261. 10.5194/hess-21-2249-2017

[B42] Pan T. Zou X. T. Liu Y. J. Wu S. H. He G. M. (2017b). Contributions of climatic and non-climatic drivers to grassland variations on the Tibetan Plateau. *Ecol. Eng.* 108 307–317. 10.1016/j.ecoleng.2017.07.039

[B43] Peng F. Xue X. You Q. Huang C. Dong S. Liao J. (2018). Changes of soil properties regulate the soil organic carbon loss with grassland degradation on the Qinghai-Tibet Plateau. *Ecol. Indic.* 93 572–580. 10.1016/j.ecolind.2018.05.047

[B44] Peng J. Liu Z. H. Liu Y. H. Wu J. S. Han Y. A. (2012). Trend analysis of vegetation dynamics in Qinghai-Tibet Plateau using hurst exponent. *Ecol. Indic.* 14 28–39. 10.1016/j.ecolind.2011.08.011

[B45] Pfisterer A. B. Schmid B. (2002). Diversity-dependent production can decrease the stability of ecosystem functioning. *Nature* 416 84–86. 10.1038/416084a 11882897

[B46] Qiu J. (2008). The third pole. *Nature* 454 393–396. 10.1038/454393a 18650887

[B47] R Development Core Team (2020). *R: A Language and Environment for Statistical Computing.* Vienna: The R Foundation for Statistical Computing.

[B48] Ran Q. W. Hao Y. B. Xia A. Q. Liu W. J. Hu R. H. Cui X. Y. (2019). Quantitative assessment of the impact of physical and anthropogenic factors on vegetation spatial-temporal variation in Northern Tibet. *Remote Sens.* 11:1183. 10.3390/rs11101183

[B49] Ren Y. J. Lu Y. H. Fu B. J. (2016). Quantifying the impacts of grassland restoration on biodiversity and ecosystem services in China: a meta-analysis. *Ecol. Eng.* 95 542–550. 10.1016/j.ecoleng.2016.06.082

[B50] Rosenberg M. S. (2005). The file-drawer problem revisited: a general weighted method for calculating fail-safe numbers in meta-analysis. *Evolution* 59 464–468. 10.1554/04-602 15807430

[B51] Ruehlmann J. Koerschens M. (2009). Calculating the effect of soil organic matter concentration on soil bulk density. *Soil Sci. Soc. Am. J.* 73 876–885. 10.2136/sssaj2007.0149

[B52] Shang Z. Long R. Ma Y. (2006). Discussion on Restoration and rebuilding of blackion soil patch’ degraded meadow in the headwater area of yangtze and yellow rivers. *Chin. J. Grassl.* 28 69–74.

[B53] Tang L. Dong S. K. Sherman R. Liu S. L. Liu Q. R. Wang X. X. (2015). Changes in vegetation composition and plant diversity with rangeland degradation in the alpine region of Qinghai-Tibet Plateau. *Rangel. J.* 37 107–115. 10.1071/rj14077

[B54] Teng Y. Zhan J. Agyemang F. B. Sun Y. (2020). The effects of degradation on alpine grassland resilience: a study based on meta-analysis data. *Glob. Ecol. Conserv.* 24:e01336. 10.1016/j.gecco.2020.e01336

[B55] Turner M. G. Smithwick E. A. H. Metzger K. L. Tinker D. B. Romme W. H. (2007). Inorganic nitrogen availability after severe stand-replacing fire in the greater yellowstone ecosystem. *Proc. Natl. Acad. Sci. U.S.A.* 104 4782–4789. 10.1073/pnas.0700180104 17360349 PMC1829215

[B56] Wang C. T. Long R. J. Wang Q. L. Jing Z. C. Shi J. J. (2009). Changes in plant diversity, biomass and soil C, in alpine meadows at different degradation stages in the headwater region of three rivers, China. *Land Degrad. Dev.* 20 187–198. 10.1002/ldr.879

[B57] Wang D. Wu G. L. Chang X. F. Shi Z. H. Sun L. Wei X. H. (2014). Higher species diversity occurs in more fertile habitats without fertilizer disturbance in an alpine natural grassland community. *J. Mt. Sci.* 11 755–761. 10.1007/s11629-013-2703-8

[B58] Weisser W. W. Roscher C. Meyer S. T. Ebeling A. Luo G. J. Allan E. (2017). Biodiversity effects on ecosystem functioning in a 15-year grassland experiment: patterns, mechanisms, and open questions. *Basic Appl. Ecol.* 23 1–73. 10.1016/j.baae.2017.06.002

[B59] Wen J. Qin R. M. Zhang S. X. Yang X. Y. Xu M. H. (2020). Effects of long-term warming on the aboveground biomass and species diversity in an alpine meadow on the Qinghai-Tibetan Plateau of China. *J. Arid Land* 12 252–266. 10.1007/s40333-020-0064-z

[B60] Wu J. Wang H. Li G. Ma W. Wu J. Gong Y. (2020). Vegetation degradation impacts soil nutrients and enzyme activities in wet meadow on the Qinghai-Tibet Plateau. *Sci. Rep.* 10:21271. 10.1038/s41598-020-78182-9 33277536 PMC7718246

[B61] Xu H. P. Zhang J. Pang X. P. Wang Q. Zhang W. N. Wang J. (2019). Responses of plant productivity and soil nutrient concentrations to different alpine grassland degradation levels. *Environ. Monit. Assess.* 19:678. 10.1007/s10661-019-7877-2 31654145

[B62] Xu T. Zhao X. Zhang X. Wang X. Geng Y. Hu L. (2020). Sustainable development of ecological grass-based livestock husbandry in Qinghai- Tibet Plateau alpine area:principle,technology and practice. *Acta Ecol. Sin.* 40 6324–6337.

[B63] Yan L. Li Y. Wang L. Zhang X. D. Wang J. Z. Wu H. D. (2020). Grazing significantly increases root shoot ratio but decreases soil organic carbon in Qinghai-Tibetan Plateau grasslands: a hierarchical meta-analysis. *Land Degrad. Dev.* 31 2369–2378. 10.1002/ldr.3606

[B64] Yu K. F. Lehmkuhl F. Falk D. (2017). Quantifying land degradation in the Zoige Basin, NE Tibetan Plateau using satellite remote sensing data. *J. Mt. Sci.* 14 77–93. 10.1007/s11629-016-3929-z

[B65] Zhang L. (2006). Exploration of ecological pitfalls embeded in the human-land interaction. *Acta Ecol. Sin.* 26 2167–2173.

[B66] Zhang Q. Liu K. S. Shao X. Q. Li H. He Y. X. Sirimuji (2021). Microbes require a relatively long time to recover in natural succession restoration of degraded grassland ecosystems. *Ecol. Indic.* 129:107881. 10.1016/j.ecolind.2021.107881

[B67] Zhang Q. Ma L. Zhang Z. Xu W. Zhou B. Song M. (2019). Ecological restoration of degraded grassland in Qinghai-Tibet alpine region: degradation status, restoration measures, effects and prospects. *Acta Ecol. Sin.* 39 7441–7451.

[B68] Zhang R. Wang X. Pu X. Xu C. Yu Y. (2015). Analysis of rangeland and livestock production systems in alpine pastoral area of Qinghai-Tibet Plateau. *J. Yunnan Agric. Univ.* 30 755–759.

[B69] Zhang W. Xue X. Peng F. You Q. Hao A. (2019). Meta-analysis of the effects of grassland degradation on plant and soil properties in the alpine meadows of the Qinghai-Tibetan Plateau. *Glob. Ecol. Conserv.* 20:e00774. 10.1016/j.gecco.2019.e00774

[B70] Zhang Y. Dong S. K. Gao Q. Z. Liu S. L. Ganjurjav H. Wang X. X. (2017). Soil bacterial and fungal diversity differently correlated with soil biochemistry in alpine grassland ecosystems in response to environmental changes. *Sci. Rep.* 7:43077. 10.1038/srep43077 28262753 PMC5338028

[B71] Zhou H. Zhang D. G. Jiang Z. H. Sun P. Xiao H. L. Wu Y. X. (2019). Changes in the soil microbial communities of alpine steppe at Qinghai-Tibetan Plateau under different degradation levels. *Sci. Total Environ.* 651 2281–2291. 10.1016/j.scitotenv.2018.09.336 30326458

[B72] Zhou Q. Chen S. Guo Z. (2016). The principles and practice of standardized demonstration ranches construction. *Chin. Sci. Bull.* 61 231–238.

[B73] Zhou Z. Wang C. Luo Y. (2020). Meta-analysis of the impacts of global change factors on soil microbial diversity and functionality. *Nat. Commun.* 11:3072. 10.1038/s41467-020-16881-7 32555185 PMC7300008

[B74] Zhou Z. Wang C. Zheng M. Jiang L. Luo Y. (2017). Patterns and mechanisms of responses by soil microbial communities to nitrogen addition. *Soil Biol. Biochem.* 115 433–441. 10.1016/j.soilbio.2017.09.015

[B75] Zhu J. T. Zhang Y. J. Liu Y. J. (2016). Effects of short-term grazing exclusion on plant phenology and reproductive succession in a Tibetan alpine meadow. *Sci. Rep.* 6:27781. 10.1038/srep27781 27301554 PMC4908376

[B76] Zhuang M. H. Gongbuzeren, Zhang J. Li W. J. (2019). Community-based seasonal movement grazing maintains lower greenhouse gas emission intensity on Qinghai-Tibet Plateau of China. *Land Use Policy* 85 155–160.

