# Supplementary material for: A Meta-Analysis on Degraded Alpine Grassland Mediated by Climate Factors: Enlightenment for Ecological Restoration
Source: Front Plant Sci. 2022 Jan 7;12:821954. doi: 10.3389/fpls.2021.821954 (PMC8777074; doi:10.3389/fpls.2021.821954)
Supplement: Supplementary file 1 [file Data_Sheet_1.docx]

Supplementary Appendix


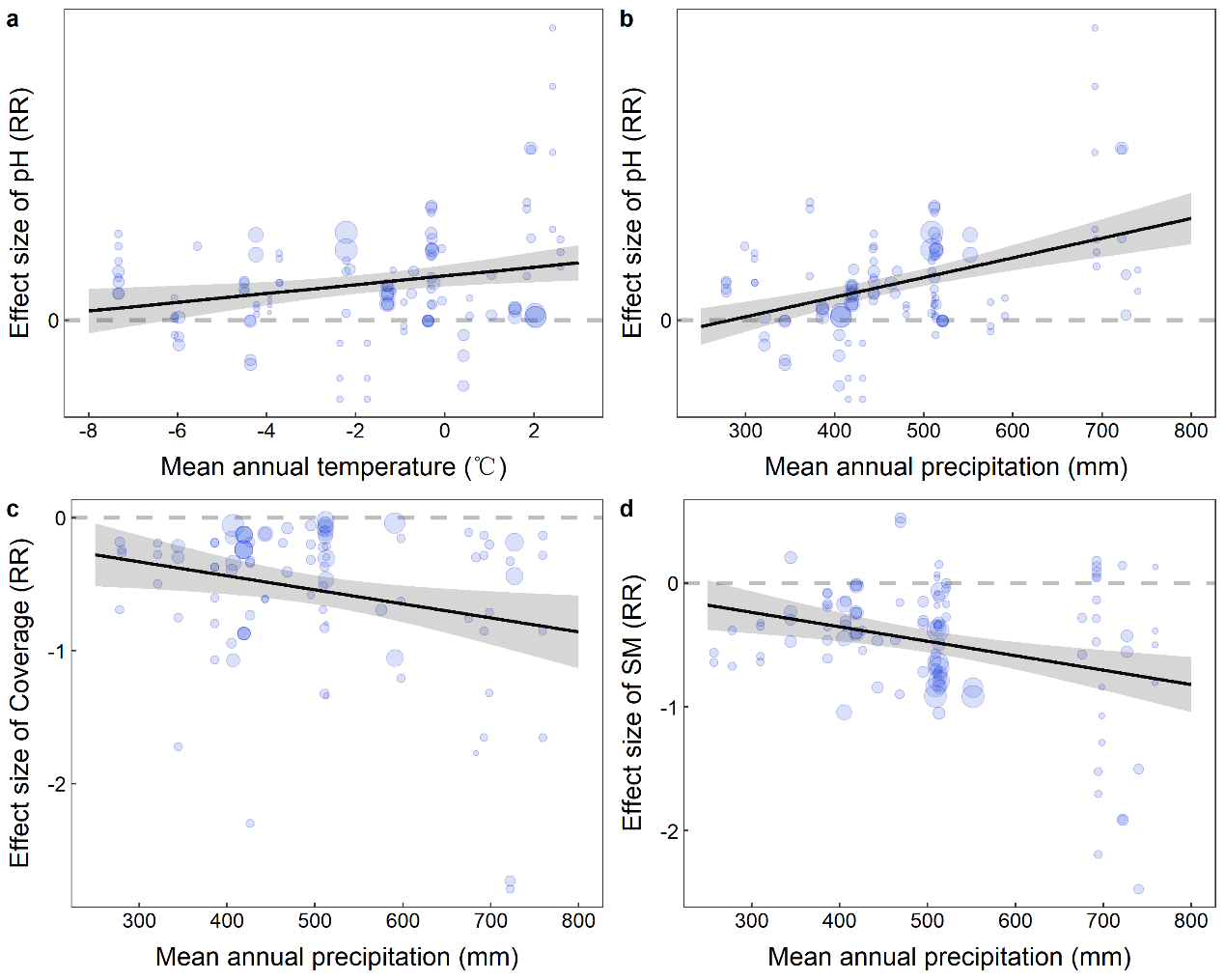


**Supplementary Figure 1** Changes in soil pH and soil moisture (SM) with climate variables. RR, response ratios. RR = 0, dashed gray line; predicted mean effect size (with 95% CI in gray), black lines. Size of data points (in blue) is proportional to the sampling variance. Results obtained with single meta-regressions.

**Supplementary Table 1** Degradation gradient of alpine grassland in the Qinghai-Tibetan Plateau (Ma et al., 2002)

| Degradation stages | Vegetation cover (%) | Grassland quality |
| --- | --- | --- |
| Non-degradation (ND) | 80-90 | Excellent |
| Light degradation (LD) | 70-85 | Good |
| Moderate degradation (MD) | 50-70 | Bad |
| Heavy degradation (HD) | 30-50 | Worse |
| Extreme degradation (ED) | < 30 | Worst |

**Supplementary Table 2** Results for publication bias. Sample size is the number of observations of a given variable. Bold types indicate significance at *P* < 0.05. A significant correlation by Egger's regression test for funnel plot asymmetry indicates possible publication bias. Rosenthal’s fail-safe number gives the number of studies needed to shift the overall effect from statistically significant to non-significant. If the Rosenthal’s fail-safe number is larger than 5n + 10 (n, number of observations), the results are robust regardless of any potential publication bias.

| **Variable** | **Sample size** | **Egger's test**  ***P* value** | **Rosenthal**  **fail-safe** |
| --- | --- | --- | --- |
| **Plant** |  |  |  |
| Vegetation cover (VC) | 94 | 0.055 |  |
| Aboveground Biomass (AGB) | 73 | 0.120 |  |
| Belowground Biomass (BGB) | 62 | 0.376 |  |
| Shannon Index | 72 | 0.541 |  |
| **Soil properties** |  |  |  |
| Bulk Density | 110 | 0.643 |  |
| Soil pH | 113 | 0.998 |  |
| Soil Moisture | 111 | 0.614 |  |
| Sand | 35 | **0.005** | 1054 |
| Silt | 32 | 0.086 |  |
| Clay | 30 | **0.005** | 40769 |
| Soil organic C (SOC) | 151 | 0.949 |  |
| Total N | 128 | **0.033** | 555768 |
| Soil C:N ratio | 33 | 0.896 |  |
| Available N | 71 | 0.317 |  |
| NH_4_^+^-N | 37 | **0.044** | 13102 |
| NO_3_^-^-N | 39 | 0.055 |  |
| Total P | 101 | 0.104 |  |
| Available P | 84 | 0.662 |  |
| Available K | 45 | 0.118 |  |
| Microbial biomass (MBC) | 27 | 0.388 |  |
| **Enzyme activities** |  |  |  |
| Oxidative C-cycling Enzymes | 20 | 0.052 |  |
| Hydrolytic C-cycling Enzymes | 36 | 0.427 |  |
| N-cycling Enzymes | 45 | 0.747 |  |
| P-cycling Enzymes | 32 | 0.447 |  |

**Supplementary Table 3** Results of the meta-regression model with MAT as moderator to explain the variance in the responses ratio (RR) of variables. Bold types indicate significance at *P* < 0.05. MAT, mean annual temperature (℃).

| **Variable** | **Slope** | **R^2^** | ***P* value** |
| --- | --- | --- | --- |
| **Plant** |  |  |  |
| Vegetation cover (VC) | 0.030 | 0.005 | 0.229 |
| Aboveground Biomass (AGB) | -0.040 | 0.028 | 0.081 |
| Belowground Biomass (BGB) | 0.019 | 0.000 | 0.619 |
| Shannon Index | 0.002 | 0.000 | 0.944 |
| **Soil properties** |  |  |  |
| Bulk Density | 0.006 | 0.000 | 0.559 |
| Soil pH | 0.009 | 0.042 | **0.007** |
| Soil Moisture | -0.017 | 0.000 | 0.404 |
| Sand | 0.071 | 0.145 | **0.015** |
| Silt | -0.123 | 0.481 | **<0.001** |
| Clay | -0.197 | 0.211 | **0.004** |
| Soil organic C (SOC) | -0.009 | 0.000 | 0.706 |
| Total N | 0.076 | 0.074 | **0.001** |
| Soil C:N ratio | -0.007 | 0.000 | 0.560 |
| Available N | 0.017 | 0.000 | 0.521 |
| NH_4_^+^-N | 0.040 | 0.004 | 0.243 |
| NO_3_^-^-N | 0.044 | 0.003 | 0.218 |
| Total P | 0.043 | 0.055 | **0.009** |
| Available P | 0.012 | 0.000 | 0.500 |
| Available K | -0.192 | 0.273 | **<0.001** |
| Microbial biomass (MBC) | -0.004 | 0.000 | 0.968 |
| **Enzyme activities** |  |  |  |
| Oxidative C-cycling Enzymes | 0.073 | 0.000 | 0.259 |
| Hydrolytic C-cycling Enzymes | 0.066 | 0.000 | 0.302 |
| N-cycling Enzymes | 0.097 | 0.024 | 0.076 |
| P-cycling Enzymes | 0.062 | 0.000 | 0.399 |

**Supplementary Table 4** Results of the meta-regression model with MAP as moderator to explain the variance in the responses ratio (RR) of variables. MAP, mean annual precipitation (mm).

| **Variable** | **Slope** | **R^2^** | ***P* value** |
| --- | --- | --- | --- |
| **Plant** |  |  |  |
| Vegetation cover (VC) | -0.001 | 0.050 | **0.014** |
| Aboveground Biomass (AGB) | -0.001 | 0.000 | 0.393 |
| Belowground Biomass (BGB) | -0.001 | 0.020 | 0.186 |
| Shannon Index | -0.001 | 0.028 | 0.106 |
| **Soil properties** |  |  |  |
| Bulk Density | 0.000 | 0.004 | 0.213 |
| Soil pH | 0.000 | 0.223 | **<0.001** |
| Soil Moisture | -0.001 | 0.089 | **0.001** |
| Sand | 0.000 | 0.000 | 0.552 |
| Silt | -0.001 | 0.180 | **0.018** |
| Clay | -0.001 | 0.000 | 0.567 |
| Soil organic C (SOC) | -0.001 | 0.007 | 0.146 |
| Total N | 0.001 | 0.054 | **0.006** |
| Soil C:N ratio | 0.000 | 0.020 | 0.150 |
| Available N | -0.000 | 0.000 | 0.471 |
| NH_4_^+^-N | 0.005 | 0.333 | **<0.001** |
| NO_3_^-^-N | 0.003 | 0.079 | 0.063 |
| Total P | 0.001 | 0.035 | **0.039** |
| Available P | 0.000 | 0.000 | 0.408 |
| Available K | 0.001 | 0.000 | 0.616 |
| Microbial biomass (MBC) | -0.003 | 0.102 | **0.046** |
| **Enzyme activities** |  |  |  |
| Oxidative C-cycling Enzymes | 0.001 | 0.000 | 0.411 |
| Hydrolytic C-cycling Enzymes | -0.003 | 0.153 | **0.013** |
| N-cycling Enzymes | 0.002 | 0.012 | 0.178 |
| P-cycling Enzymes | -0.004 | 0.188 | **0.006** |

**Supplementary Table 5** Results of the meta-regression model with non-degradation variables as moderator to explain the variance in the responses ratio (RR) of variables.

| **Variable** | **Slope** | **R^2^** | ***P* value** |
| --- | --- | --- | --- |
| AGB | -0.001 | 0.130 | **<0.001** |
| BGB | -0.000 | 0.000 | 0.761 |
| SOC | -0.002 | 0.036 | **0.015** |
| TN | -0.009 | 0.024 | **0.049** |
| MBC | -0.000 | 0.000 | 0.846 |
